# Vesicular Stomatitis Virus Chimeras Expressing the Oropouche Virus Glycoproteins Elicit Protective Immune Responses in Mice

**DOI:** 10.1128/mBio.00463-21

**Published:** 2021-08-03

**Authors:** Sarah Hulsey Stubbs, Marjorie Cornejo Pontelli, Nischay Mishra, Changhong Zhou, Juliano de Paula Souza, Rosa Maria Mendes Viana, W. Ian Lipkin, David M. Knipe, Eurico Arruda, Sean P. J. Whelan

**Affiliations:** a Department of Microbiology, Harvard Medical School, Boston, Massachusetts, USA; b Department of Molecular Microbiology, Washington University School of Medicine in St. Louis, Saint Louis, Missouri, USA; c Center for Infection and Immunity, Mailman School of Public Health, Columbia University, New York, New York, USA; d Department of Cell and Molecular Biology, Virology Research Center, Ribeirao Preto School of Medicine, University of São Paulo, Ribeirao Preto, São Paulo, Brazil; University of Cambridge; Icahn School of Medicine at Mount Sinai

**Keywords:** Oropouche virus, arbovirus, bunyavirus, emerging infectious diseases, vaccines

## Abstract

Oropouche virus (OROV) infection of humans is associated with a debilitating febrile illness that can progress to meningitis or encephalitis. First isolated from a forest worker in Trinidad and Tobago in 1955, the arbovirus OROV has since been detected throughout the Amazon basin with an estimated 500,000 human infections over 60 years. Like other members of the family *Peribunyaviridae*, the viral genome exists as 3 single-stranded negative-sense RNA segments. The medium-sized segment encodes a viral glycoprotein complex (GPC) that is proteolytically processed into two viral envelope proteins, Gn and Gc, responsible for attachment and membrane fusion. There are no therapeutics or vaccines to combat OROV infection, and we have little understanding of protective immunity to infection. Here, we generated a replication competent chimeric vesicular stomatitis virus (VSV), in which the endogenous glycoprotein was replaced by the GPC of OROV. Serum from mice immunized by intramuscular injection with VSV-OROV specifically neutralized wild-type OROV, and using peptide arrays we mapped multiple epitopes within an N-terminal variable region of Gc recognized by the immune sera. VSV-OROV lacking this variable region of Gc was also immunogenic in mice producing neutralizing sera that recognize additional regions of Gc. Challenge of both sets of immunized mice with wild-type OROV shows that the VSV-OROV chimeras reduce wild-type viral infection and suggest that antibodies that recognize the variable N terminus of Gc afford less protection than those that target more conserved regions of Gc.

## INTRODUCTION

Oropouche virus (OROV), first isolated in 1955 from a forest worker in Trinidad and Tobago, causes a debilitating febrile illness in humans that can progress to meningitis or encephalitis (1, 2). OROV is the most prevalent arbovirus after dengue in Brazil (3, 4) and is currently circulating in Argentina, Bolivia, Colombia, Ecuador, and Venezuela (5–7). In urban areas across the Amazon region, seroprevalence rates of up to 15 to 33% suggest that OROV infection is underappreciated (8, 9). The virus infects a broad range of species and has both a sylvatic and an urban cycle. During the sylvatic cycle, the virus infects sloths, monkeys, rodents, and birds, with Coquillettidia venezuelensis and Ochlerotatus serratus serving as vectors (1). In the urban cycle, Culicoides paraensis and Culex quinquefasciatus serve as vectors of OROV (1), with human infections paralleling increases in the vector population during the rainy season (10). Infection is predominantly seen in individuals returning from forested areas (11), with limited evidence of direct human to human transmission (12). Climate change, expansion and dissemination of vectors, globalization of travel, and habitat loss likely contribute to increases in OROV infections (13). Despite the growing dissemination of OROV, and the risks posed by this emergent threat, there are currently no therapeutics to combat OROV infection.

Oropouche virus, a member of the family *Peribunyaviridae*, contains a single-stranded, negative-sense RNA genome divided on three segments providing a total genome size of 11,985 nucleotides. The large segment (l) encodes the large protein (L), which acts as the RNA-dependent RNA polymerase, the medium segment (m) encodes the viral glycoprotein complex (GPC), and the small segment (s) encodes the nucleocapsid protein that sheaths the genomic and antigenomic RNA. Two nonstructural proteins, NSs and NSm are coded by the small and medium segments, respectively. The GPC is synthesized as a single polyprotein that undergoes cotranslational cleavage by host-cell proteases into the two glycoproteins, Gn and Gc, and liberates the intervening NSm protein (14). The Gc protein acts as the viral fusogen (15), and Gn mediates attachment and shields and protects Gc from premature triggering (16). Extrapolating from studies with La Crosse (17–20) and Schmallenberg (16, 21) viruses, both in the same orthobunyavirus genus of the *Peribunyaviridae*, antibodies against the glycoproteins may protect against infection and are therefore an important objective of vaccine strategies against OROV.

Vesicular stomatitis virus (VSV), the prototype of the family *Rhabdoviridae*, infects cells by a single attachment and fusion glycoprotein (G) (22). The development of reverse genetic approaches to manipulate the VSV genome (23) permitted replacement of the attachment and fusion machinery with those of heterologous lipid enveloped viruses (24–28). In the case of Zaire ebolavirus, the resulting VSV-ZEBOV chimera is a live attenuated vaccine, ERVEBO, that is approved for use in humans (29–31). Analogous vaccine candidates are in preclinical and clinical development for multiple enveloped viruses (25, 27, 32), including severe acute respiratory syndrome 2 coronavirus (33). In addition to providing a platform for development of candidate vaccines, the VSV-chimeric viruses have also permitted investigation of viral tropism and cellular entry pathways and helped us understand correlates of immune protection (30, 34–38).

Building on this proven approach, we generated a VSV-chimera in which the native glycoprotein gene is replaced by the GPC of OROV. The resulting VSV-OROV chimera replicates to high titers in BSRT7 cells in culture and efficiently incorporates the Gn and Gc of OROV into particles. Following single-dose or prime-boost intramuscular injection of mice, VSV-OROV elicits the production of immune-specific sera that neutralize wild-type OROV. Using peptide arrays corresponding to the OROV glycoproteins, we find significant reactivity to the more variable N-terminal domain of Gc that precedes the domains that are structurally homologous to other class II fusion proteins. We generated two additional VSV-OROV chimeras lacking portions of the variable N terminus of Gc, termed the “head” and “stalk” domains. Although both variants yielded replication competent virus, deletion of the stalk domain led to the accumulation of mutations in Gc. Immunization of mice with the VSV-OROV lacking the head domain of Gc generated immune sera reactive with new regions of Gc. Challenge studies demonstrate that immunization with VSV-OROV or the chimera lacking the head domain of Gc offers protection against wild-type OROV infection as evidenced by weight loss, temperature, and viral burden. This study demonstrates that VSV-OROV immunization induces neutralizing serum responses that can protect mice against challenge with wild-type OROV.

## RESULTS

### Construction and characterization of VSV-Oropouche chimeras.

**FIG 1:**
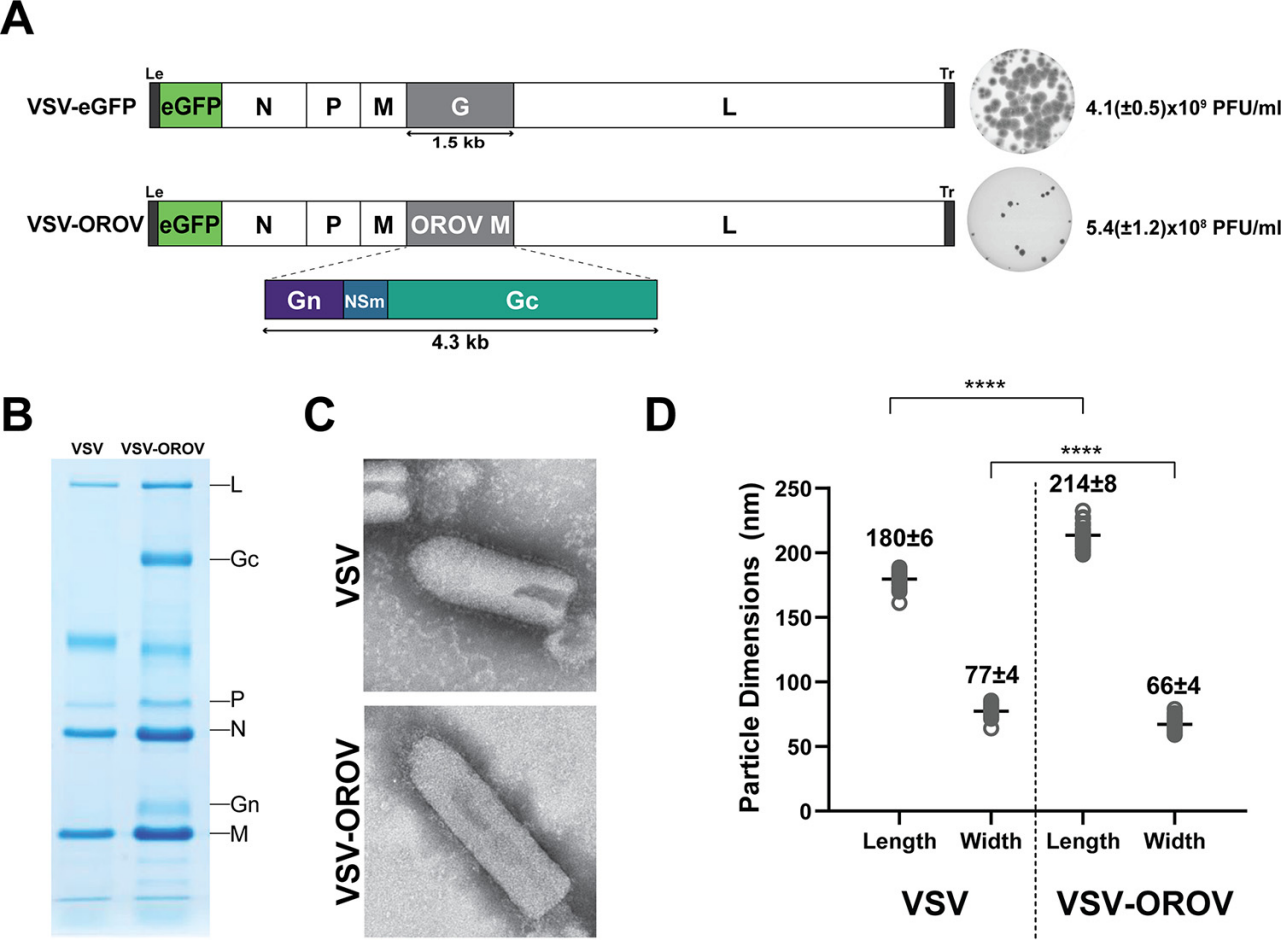
Generation and characterization of VSV-OROV. (A) Genomic organization of VSV-eGFP and VSV-OROV. Viral genomes are shown in the 3′ to 5′ orientation. The five viral genes, N (nucleocapsid), P (phosphoprotein), M (matrix), G (glycoprotein), and L (large polymerase), are shown. Enhanced green fluorescent protein (eGFP) is located in the first position and serves as a marker for infection. The VSV glycoprotein was replaced with the gene encoding the OROV M segment to generate VSV-OROV. Representative plaque assays are shown on the right as well as the endpoint titers in BSRT7 cells. (B) SDS-PAGE analysis of purified virions stained with Coomassie blue. Viral proteins are indicated on the right. (C) Electron micrographs of purified virions stained with 2% PTA. (D) Measurements of individual viral particles were carried out from micrographs like those shown in panel C. Each circle represents a single virion, and the line denotes the mean with the standard deviation (*n *= 35). Length and width differences between VSV and VSV-OROV particles were statistically significant; ****, *P* < 0.0001.

Orthobunyavirus virions incorporate two viral glycoproteins on their surface, Gn and Gc, that mediate binding and entry into the cell. Gn and Gc are synthesized as part of a polyprotein precursor GPC. We engineered an infectious molecular clone of VSV that expresses enhanced green fluorescent protein (eGFP) as a marker of infection (39), to replace the native attachment and fusion glycoprotein with the GPC polyprotein of OROV (Fig. 1A). Using established procedures, we recovered a chimeric virus, VSV-OROV, capable of autonomous replication as evidenced by plaque formation (Fig. 1A). Kinetic analysis of the yields of infectious VSV-OROV identify a 1-log reduction in viral growth compared to VSV-eGFP (Fig. 1A). Analysis of the protein composition of purified virions by SDS-PAGE demonstrates that Gn and Gc are incorporated into VSV-OROV in place of VSV G (Fig. 1B). Mass spectrometry analysis of the band above P in the VSV-OROV sample identified the band as an albumin contaminant. VSV-OROV particles retain the classic bullet shape of VSV as evidenced by negative-stain electron microscopy and are visually decorated with spikes consistent with incorporation of the OROV glycoproteins (Fig. 1C). As insertion of the M open reading frame (ORF) of OROV increases the genome length of VSV by approximately 2,500 nucleotides, the VSV-OROV particles are approximately 34 nm longer than VSV particles and 11 nm narrower (Fig. 1D).

### Effect of truncation of the OROV Gc N-terminal variable region on viral infectivity.

**FIG 2:**
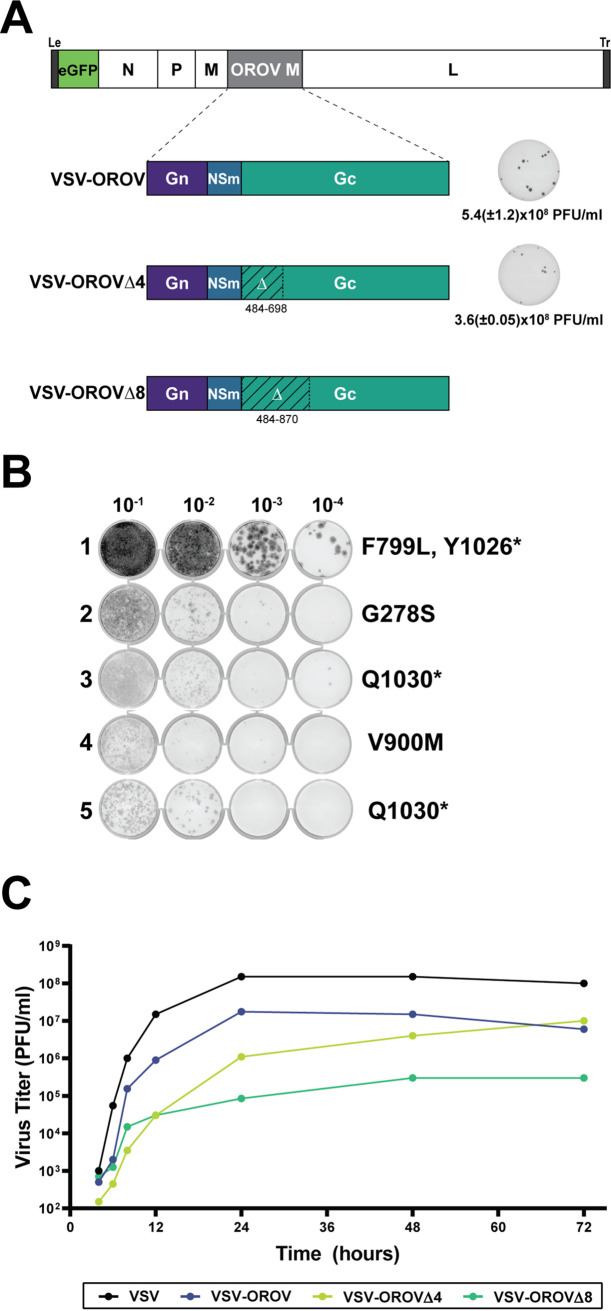
Generation of VSV-OROV chimeras and mutants. (A) Genomic organization of VSV-OROV, VSV-OROVΔ4, and VSV-OROVΔ8. Amino acids 484 to 698 were deleted from the N-terminal region of Gc to generate VSV-OROVΔ4, and amino acids 484 to 870 were deleted to generate VSV-OROVΔ8. Plaque assays of VSV-OROV (shown also in Fig. 1) and VSV-OROVΔ4 on BSRT7 cells are shown on the right. (B) VSV-OROVΔ8 isolates were passaged on BSRT7 cells, and the M segments from 5 isolates were sequenced by Sanger sequencing. Mutations found in the M segment are denoted on the right. (C) Growth curve of VSV, VSV-OROV, VSV-OROVΔ4, and VSV-OROVΔ8 Q1030*.

For the orthobunyavirus, bunyamwera (BUNV), the N-terminal half of Gc is dispensable for viral replication in cell culture (40). Phylogenetic analysis suggests the N terminus of Gc comprises two variable domains that have novel folds, and recent structural studies reveal that those variable domains correspond to a head and stalk domains of Gc (16). Deletion of the head domain results in enhanced cell fusion for BUNV, suggesting that this region may act to mask or protect the highly conserved fusion peptide (40). Elimination of the head and stalk domain resulted in a further reduction in viral yield, although the mechanism underlying this is unclear (40). Using the infectious cDNA of VSV-OROV, we engineered the analogous variants lacking the head domain of Gc (VSV-OROVΔ4) or the head and stalk domains (VSV-OROVΔ8). Autonomously replicating viruses were recovered from both variants (Fig. 2A). Sequence analysis demonstrates that no other changes were present in the GPC gene of VSV-OROV or VSV-OROVΔ4, but each of the VSV-OROVΔ8 isolates sequenced contained additional mutations in Gc (Fig. 2B), with three of the variants containing premature stop codons in the Gc cytoplasmic tail. Although growth of both truncated variants was attenuated compared to VSV-OROV, VSV-OROVΔ4 reached comparable titers (Fig. 2C). This result demonstrates that as for BUNV, at least a portion of the N terminus of Gc is dispensable for infectivity of cells mediated by the glycoprotein complex of OROV.

### VSV-OROV chimeras elicit production of neutralizing antibodies in mice.

**FIG 3:**
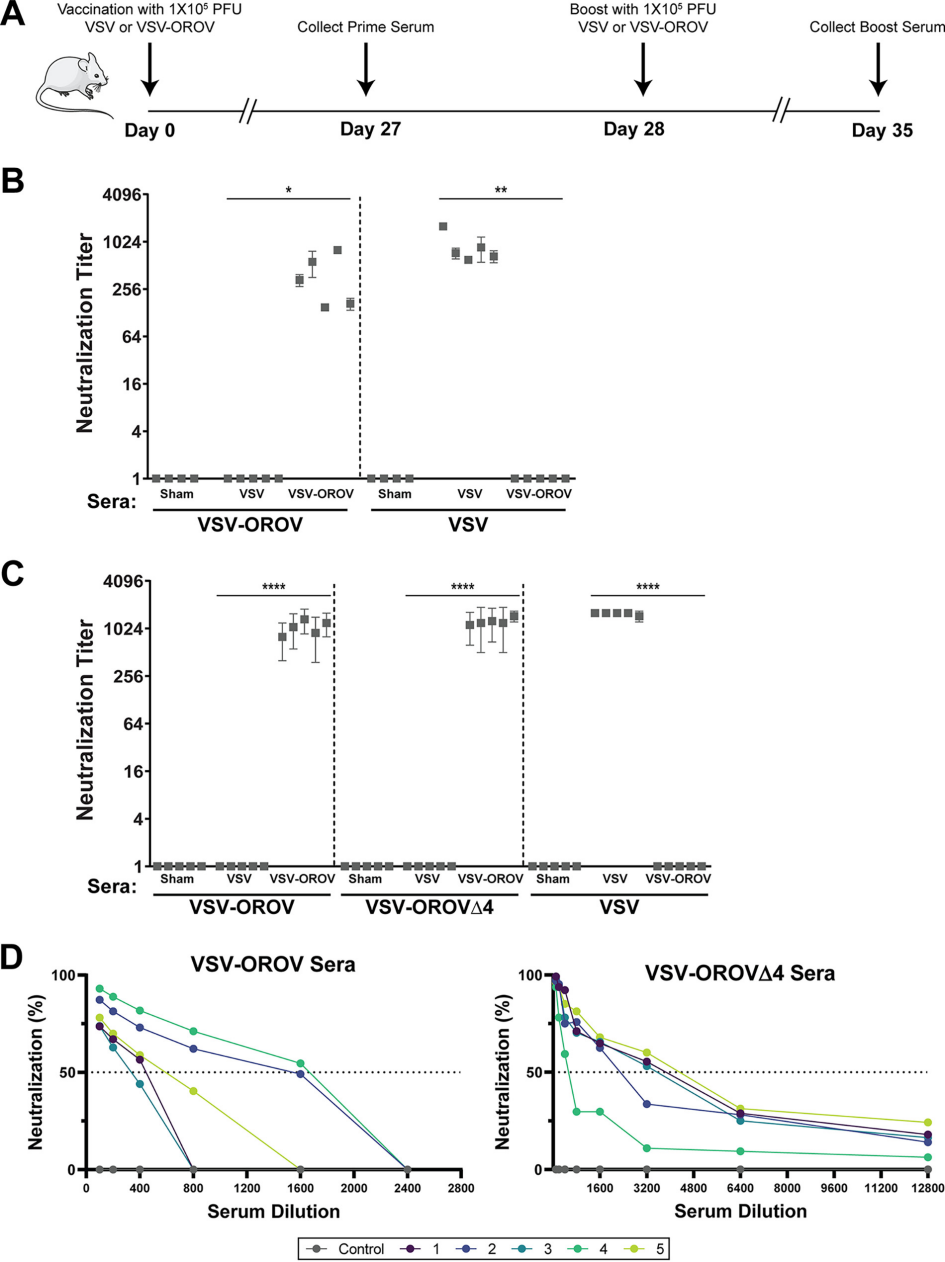
Inoculation of mice with VSV recombinants bearing OROV glycoproteins generates neutralizing antibodies. (A) Inoculation schedule of BALB/c mice. Animals (*n* = 5 per group) were immunized intramuscularly with VSV-eGFP, VSV-OROV, or VSV-OROVΔ4 and then boosted on day 28. Mice were sacrificed on day 35, and serum was collected. (B) Reciprocal dilutions of VSV or VSV-OROV serum that protects cells from 100 TCID_50_ units of indicated viruses are shown (*n *= 3). (C) Reciprocal dilution of VSV or VSV-OROVΔ4 serum that protects cells from the viruses indicated (*n *= 3). No neutralization at the highest concentration of sera (1:100) was scored as 1. Statistical analysis was performed using an unpaired *t* test (*, *P* < 0.05; **, *P* < 0.01; ****, *P* < 0.0001). (D) Plaque reduction neutralization assays were performed to assess neutralization of wild-type OROV. Plaques were visually scored after incubation with serial dilutions of mouse serum. Each line (numbered 1 to 5) represents an individual mouse.

To determine whether the VSV-OROV chimeras were immunogenic, we inoculated BALB/c mice intramuscularly with VSV or the indicated VSV-OROV chimera, boosted the animals 28 days later, and examined serum-neutralizing titers at days 27 and 35 postinoculation (Fig. 3A). Inoculation of mice with VSV-OROV generates sera that specifically neutralize VSV-OROV but not VSV, as measured by cell culture infection assays. Correspondingly, sera from mice inoculated with VSV specifically neutralize VSV but not VSV-OROV, validating that neutralization depends on the surface glycoproteins and not other components of the virion (Fig. 3B). All mice produced antibodies capable of neutralizing the relevant virus, whereas sera from sham-vaccinated animals failed to neutralize either VSV-OROV or VSV. Mice inoculated with VSV-OROVΔ4 generated sera that neutralized both VSV-OROV and VSV-OROVΔ4 (Fig. 3C). The increased sensitivity of VSV-OROVΔ4 to neutralization suggests loss of the head domain may increase accessibility of important neutralizing epitopes.

To examine the ability of the mouse sera to neutralize wild-type OROV, we tested serum samples from the 5 individual mice using a plaque reduction neutralization titer assay (Fig. 3D). Consistent with the ability of the sera to neutralize the respective VSV-chimeras, mice that generated neutralizing antibodies against VSV-OROV neutralized wild-type OROV, showing 50% decreases in infectivity at serum dilutions of >1:200 to 1:1,600. Consistent with the observed neutralization of the VSV-chimeras, sera from mice inoculated with VSV-OROVΔ4 were more potent than those inoculated with VSV-OROV, and serum from each mouse exhibited a >50% inhibition of infection at dilutions of 1:400 to 1:3,200 (Fig. 3D). We conclude that VSV-OROV results in production of sera that neutralize wild-type virus and that deletion of the N-terminal portion of Gc further increases neutralizing titers, perhaps by targeting additional epitopes.

### VSV-OROV and VSV-OROVΔ4 sera generate antibodies to both Gn and Gc.

**FIG 4:**
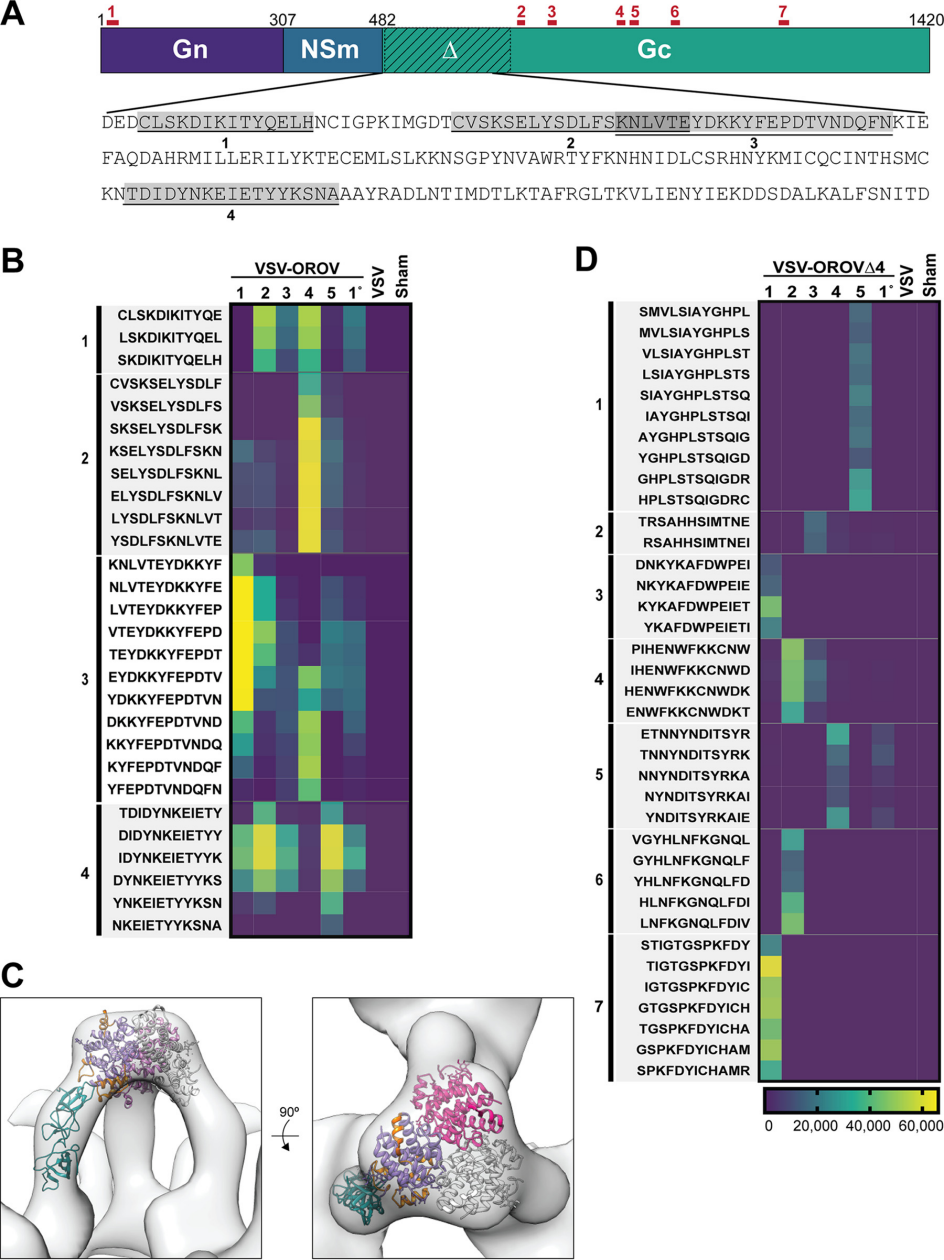
VSV-OROV and VSV-OROVΔ4 antibodies target Gn and Gc. (A) Schematic of the OROV M segment showing the two glycoproteins (Gn and Gc) as well as the nonstructural, NSm protein. Peptides identified in the peptide array with VSV-OROV are indicated in gray boxes, while peptides identified in the VSV-OROVΔ4 peptide array are indicated above the schematic in red lines and numbers. (B) Heat map of the VSV-OROV peptide array. Analysis of the peptide array was performed with five VSV-OROV mouse serum samples, one primary bleed sample, one VSV serum sample, and a sham control. Scale is shown in arbitrary units. (C) Mapping of the four identified peptides to the structure of the OROV Gc N-terminal head domain that was determined by Hellert et al. (16) (D) Peptide array from VSV-OROVΔ4 sera, like what is shown in panel B for VSV-OROV.

To identify epitopes recognized by the serum from each immunized mouse, we utilized peptide arrays designed to detect antibodies targeting linear epitopes. The tiled array is composed of ∼170,000 nonredundant 12-mer peptides from seven arboviruses, including OROV (41), and is formatted in a sliding window pattern such that there is an 11-amino acid overlap along the peptide sequence of each virus. The peptides are randomly distributed across the array to prevent location bias. This peptide array was previously used to diagnose Zika virus infection and identified a highly specific Zika epitope within the NS2B protein (41). Profiling of the sera from each mouse inoculated with VSV-OROV identified 4 peptides in a 150-residue region of the ectodomain of Gc, with two of the peptides overlapping by 6 amino acids (Fig. 4A and B). This region of Gc corresponds to the N-terminal head domain of the variable region of Gc (16) (Fig. 4C).

Mice immunized with VSV-OROVΔ4, lacking the N-terminal region of Gc, generated immune sera that reacted with linear epitopes that target both Gn and Gc of OROV. Specifically, we identified one peptide at the N terminus of Gn and six epitopes distributed throughout Gc. Five of the six Gc peptides are located N-terminal to the fusion peptide, with 1 peptide mapping approximately 100 residues to the C terminus of the fusion peptide. No conserved peptides were identified across both arrays. Earlier work with Bunyamwera virus defined the C-terminal half of Gc as important for cell fusion and for interaction of Gc and Gn for trafficking through the Golgi (40). When aligned with Gc, two of the five peptides, 974 to 989 and 1159 to 1176, map to the C-terminal half of Gc. These data demonstrate that removal of the N-terminal head domain of Gc does not impact the ability of mice to generate neutralizing sera to VSV-OROV and that linear epitopes are generated that target both Gn and Gc.

### Effect of immunization with VSV-OROV chimeras on challenge with wild-type OROV.

**FIG 5:**
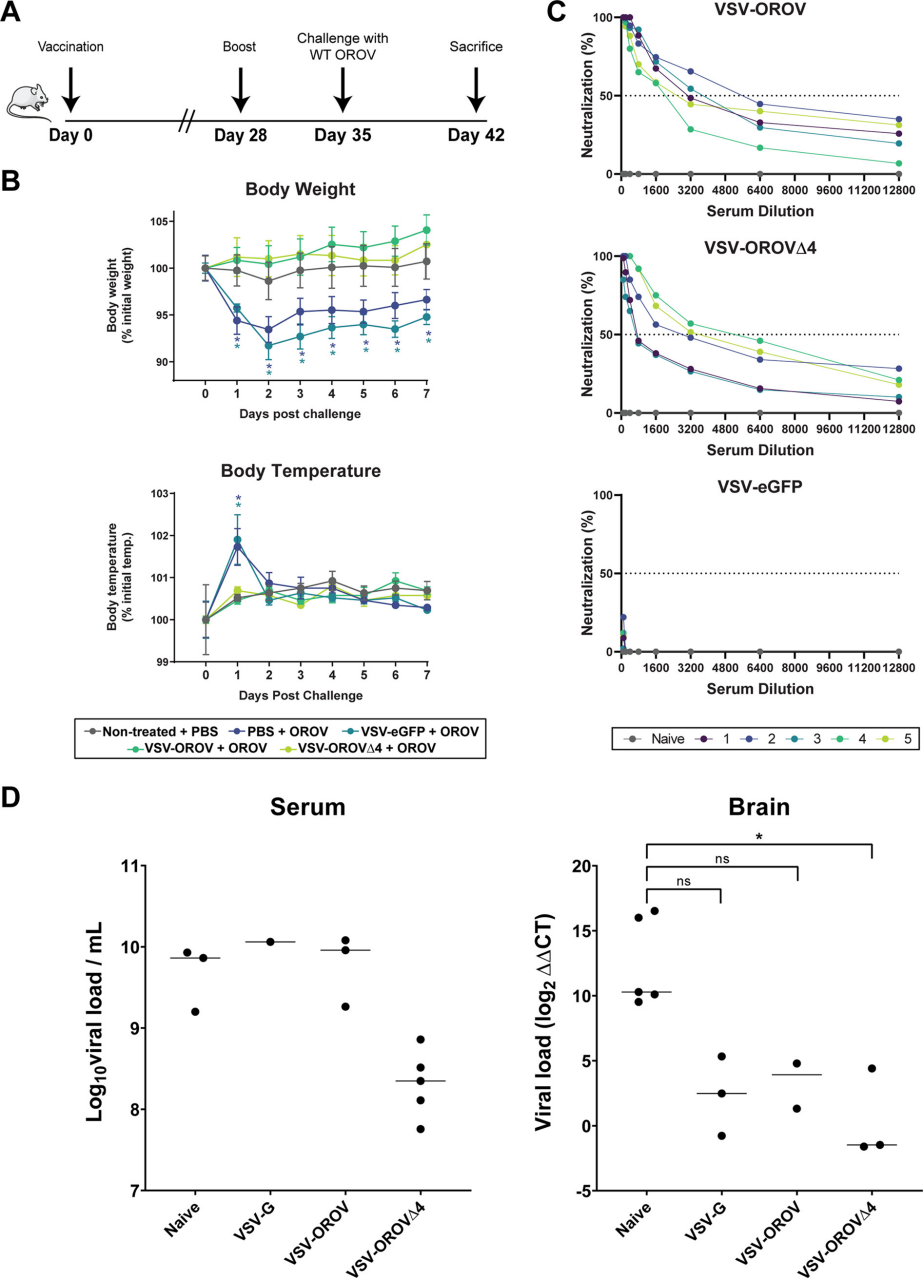
Prime-boost vaccination of mice with VSV recombinants and challenge with OROV. (A) Inoculation schedule of male, 6-week-old C57BL/6 mice. Mice were inoculated on day zero with 10^6^ FFU, boosted on day 28 with the same dose, and then challenged 1 week later with 10^6^ TCID_50_ OROV (five mice per group). One week after challenge, mice were sacrificed. (B) Body weight (top) and temperature (bottom) were assessed each day following challenge with OROV. *, *P* < 0.001. (C) One day before challenge, VSV-OROV, VSV-OROVΔ4, and VSV sera were collected from mice and tested for neutralization of OROV. Each line (numbered 1 to 5) represents an individual mouse. (D) Viral loads were assessed by measuring S segment copies in blood and brain samples from mice following sacrifice. Brain samples were normalized to the internal control, HRPT1. *, *P* < 0.05.

To determine whether VSV-OROV can elicit a protective immune response, we employed a 2-dose prime-boost immunization regimen followed by a wild-type virus challenge. Briefly, groups of mice (*n* = 5) received intramuscular inoculation of 10^6^ infectious units of VSV, VSV-OROV, or VSV-OROVΔ4 and an equivalent immunization 28 days later. Animals were challenged 7 days later with 10^6^ 50% tissue culture infective dose (TCID_50_) of wild-type virus through subcutaneous injection, which most closely simulates insect bites, and their weights and temperatures were examined daily. At 7 days postchallenge, the levels of viral RNA in the blood and brain were examined using a quantitative reverse transcription PCR (qRT-PCR) assay (Fig. 5A). Mice immunized with VSV or given phosphate-buffered saline (PBS) as a sham lost 5 to 10% body weight and exhibited a spike in body temperature following challenge. In contrast, animals immunized with VSV-OROV or VSV-OROVΔ4 continued to increase in body weight postchallenge and were not febrile (Fig. 5B). We confirmed that mice immunized with this 2-dose regimen of the VSV-OROV chimeras have high neutralizing serum titers against OROV 1 day prior to challenge, whereas the VSV- or sham-vaccinated animals had no detectable neutralizing titers (Fig. 5C).

To examine the extent of protection against infection upon challenge, we examined viral S segment RNA levels by qRT-PCR 7 days postchallenge. We detected S segment RNA copies in the blood of all groups of challenged animals, although levels were lowest in the VSV-OROVΔ4-immunized mice. This result demonstrates that the animals develop a viremia following challenge with wild-type virus in the face of a neutralizing serum response (Fig. 5D). While both VSV-OROV- and VSV-OROVΔ4-immunized mice had reduced viremia compared to VSV-immunized mice, VSV-OROVΔ4 viremia was reduced almost 3-fold compared to VSV-OROV-immunized mice. Analysis of brain tissue demonstrates that mice immunized with VSV-OROVΔ4 have reduced viral RNA levels compared with naive mice (Fig. 5D), consistent with reduced OROV infection. Analysis of individual mouse viral loads showed that mice immunized with VSV-OROV or VSV-OROVΔ4 had decreased levels of viral RNA in brain tissues compared with blood samples, while naive and VSV-vaccinated mice did not show this trend.

## DISCUSSION

We report three VSV-chimeric viruses expressing the surface proteins Gn and Gc of the emerging orthobunyavirus, Oropouche, and evaluate those chimeras for immunogenicity and protection against challenge in a mouse model of disease. The VSV chimeras are highly immunogenic in mice, yielding serum-neutralizing titers of up to 1:6,400 against wild-type OROV following a 2-dose intramuscular immunization regimen. Using overlapping peptide arrays, we demonstrate that neutralizing sera include antibodies that recognize peptides in the highly variable N terminus of Gc and demonstrate that elimination of this region results in sera that recognize more conserved regions of Gc. Challenge studies demonstrate that immunized mice are infected, although they exhibit reduced clinical symptoms and viral loads. This work demonstrates that neutralizing antisera offer some protection against Oropouche infection and provides new biosafety level 2 (BSL2) tools that can be used to understand the functions of Gn and Gc.

The spread of Oropouche virus in the Amazon region has prompted efforts to advance countermeasures against infection. The IMP dehydrogenase inhibitor, ribavirin, has no efficacy against OROV (42), and although innate immune responses control infection of mice by OROV (43), interferon alpha (IFN-α) treatment offers only limited protection of mice when administered within 3 h of infection (42, 44). Earlier efforts to develop vaccines for OROV have included deletion of the NSs and NSm proteins in infectious molecular clones of OROV (3). A similar strategy shows promise for Schmallenberg virus (SBV), an orthobunyavirus that infects ruminants (45). Although correlates of immunity are not completely understood for many bunyaviruses, vaccines targeting the glycoproteins appear to illicit strong immune responses for at least some bunyaviruses. DNA vaccines expressing Gn and Gc protected mice from challenge with Rift Valley fever virus (RVFV), while Gn and Gc of Crimean-Congo hemorrhagic fever orthonairovirus (CCHFV) were not sufficient to confer protection (46). Subunit vaccines comprising the head domain of the orthobunyavirus SBV Gc protected mice from clinical symptoms of disease, while a misfolded version of the head domain did not (47).

Here, we demonstrate that immunization of mice with VSV-OROV chimeras generates a strong neutralization serum response against wild-type OROV and that neutralizing serum response correlates with some protection against infection as evidenced by reduced clinical signs—such as weight loss and a febrile response—although viral infection was not prevented. Mice remained viremic despite immunization; however, viral burden in brain samples was decreased. One hypothesis for this result is that while the vaccines do not appear to be fully protective, they do limit the transmission of virus across the blood-brain barrier. Although the VSV-OROV constructs appear to be somewhat protective against wild-type OROV infection, incomplete inhibition suggests that a critical component may be missing.

Analysis of the serum-neutralizing antibody response using linear peptide arrays identified four prominent epitopes within the N-terminal region of Gc. All four of those peptides lie within the head domain of the Gc N-terminal region (16). Recognition of the OROV head domain fits with earlier work on La Crosse virus (LACV) and SBV that has also shown that a large proportion of glycoprotein-specific antibodies are targeted to the Gc head domain (20, 21) and supports the hypothesis that the head domain of Gc shields the functionally critical membrane fusion machinery from the antibody response and thus limits antigenic drift of the core fusion machinery. Immunization of mice with the purified head or head and stalk domains of Gc protects against infection with SBV (16, 47), demonstrating that for some orthobunyaviruses, targeting this region of Gc may be an effective strategy for vaccine development. Although we did not directly test whether the N terminus of Gc of OROV can elicit the induction of protective immune responses alone, its loss did not diminish neutralizing serum titers and instead led to the identification of additional epitopes within Gc. Though we observe a modest distinction in the extent of infection upon challenge of mice immunized with VSV-OROV and VSV-OROVΔ4, we do not understand the basis for this distinction. Development of Oropouche vaccines will require a deeper understanding of correlates of immune protection, and whether immune protection is achieved following natural infection. Indeed, while most neutralizing antibodies identified to date have targeted bunyaviral glycoproteins, bunyaviral nucleoproteins have also been shown to be highly immunogenic (48–51). Targeting the nucleoprotein has proved to be an effective method for generating protection in other RNA viruses through both B and T cell-mediated immunity, and it will be important to test whether OROV N can also provide a boost in protection. The VSV-OROV chimeras described here may aid in this analysis by providing useful BSL2 tools to study Gn and Gc function and their inhibition.

## MATERIALS AND METHODS

### Cell lines.

African green monkey Vero cells and BSRT7 Syrian golden hamster kidney cells (generously provided by K. Conzelmann) (52) were maintained at 37°C and 5% CO_2_ in Dulbecco’s modified Eagle medium (DMEM) supplemented with 10% fetal bovine serum (FBS).

### VSV recombinant generation, growth, purification, and titration.

The entire M segment of OROV BeAn19991 was codon optimized for human cell expression and synthesized using GenScript (Piscataway, NJ). The OROV M segment was cloned into pVSV-ΔG-eGFP (39) using the MluI and NotI sites. To generate OROVΔ4, site-directed mutagenesis was performed using primers GATATCAACCTGGGCAGG and CTCGTCGGCGTACACTGT. VSV-OROV and VSV-OROVΔ4 were generated by transfecting the genomic plasmid along with plasmids containing VSV N, P, L, and G into BSRT7 cells, as previously described (23). VSV containing enhanced green fluorescent protein (eGFP) was previously generated (39). All viruses were grown on BSRT7 cells in DMEM containing 2% FBS and penicillin-streptomycin/kanamycin. The titers of viral supernatants were determined by plaque assay as previously described (53), or the supernatants were gradient-purified on a 15 to 45% sucrose gradient and then the titers were determined. GFP-expressing plaques were visualized on a Typhoon 9400 imager. Purified virus was run on a 10% (wt/vol) acrylamide 0.13% (wt/vol) bis-acrylamide gel. Gels were stained with Coomassie reagent.

### Electron microscopy.

VSV and VSV-OROV were deposited onto carbon-coated copper grids and then stained with 2% (wt/vol) phosphotungstic acid (PTA) in H_2_O (pH 7.5). A Technai G^2^ Spirit BioTwin transmission electron microscope (FEI, Hillsboro, OR) was used to visualize particles. The length and width of viral particles was measured using ImageJ.

### Generation of neutralizing sera.

Six-week old male BALB/c mice (Taconic Farms) were injected intramuscularly (IM) with 10^5^ PFU of VSV, VSV-OROV, VSV-OROVΔ4, or sham on day 0. On day 27, tail vein blood samples were collected, and on day 28, mice were boosted with an additional dose of 10^5^ PFU of virus. Then, 7 days later, on day 35, mice were sacrificed, and blood samples were collected by cardiac puncture. Serum was separated by centrifugation at 1,500 × *g* for 10 min at room temperature and then heated at 56°C for 30 min to inactivate complement.

### Serum neutralization assays.

Heat-inactivated serum samples from mice were diluted in DMEM in 2-fold dilution series ranging from 1:100 to 1:1,600 in duplicate on a 96-well plate. Then, 100 TCID^50^ units of VSV, VSV-OROV, or VSV-OROVΔ4 were incubated with each serum dilution in wells for 1.5 h at 37°C. Following incubation of virus and serum, 30,000 BSRT7 cells were added to each well and incubated at 34°C for 48 h. Then, 96-well plates were scanned on a Typhoon FLA9500 imager, and dilutions lacking any GFP signal were recorded. Neutralization assays were performed three times.

### OROV sera peptide arrays.

VSV, VSV-OROV, VSV-OROVΔ4, and sham serum samples were analyzed using a previously described arboviral peptide array (41, 54).

### WT OROV stock production.

OROV strain BeAn19991 was originally donated by Luiz Tadeu Figueiredo and propagated by serial passages in Vero cells by routine methods using DMEM. Virus titration was performed by plaque assay in Vero cells plated at 4 × 10^4^ cells/well in 48-well plates 1 day prior to infection. After 1 h of incubation with virus, cells were replenished with DMEM supplemented with 2% vol/vol FBS, 1% vol/vol antibiotics, and 1% vol/vol carboxymethyl cellulose (CMC) (Sigma-Aldrich) and incubated at 37°C and 5% CO_2_ for 4 days. Cells were fixed with 4% formaldehyde for 20 min at room temperature, washed in phosphate buffered saline (PBS) (Gibco), and stained with 20% vol/vol ethanol-violet crystal solution for 15 min.

### OROV PRNT_50_.

The titer of neutralizing antibodies was determined on serum obtained on day 28 after immunization with VSV, VSV-OROV, or VSV-OROVΔ4 mice by a standard plaque reduction neutralization assay. Plaques were scored visually after incubation with serial dilutions of the mouse serum, and the 50% plaque reduction neutralization titer (PRNT_50_) was determined (55).

### Immunization and OROV challenge.

A total of 25 male C57BL/6 6-week-old mice were assigned to 5 groups; two sham groups were administered sterile 1× PBS, and three groups were immunized with VSV, VSV-OROV, or VSV-OROVΔ4. Injections were made subcutaneously in the dorsal lumbar area with 10^6^ focus-forming units (FFU) in a volume of 50 μl. At 28 days postvaccination, a booster immunization was administered to animals with the same virus dose or volume of PBS. One week later, the VSV recombinant-immunized and one sham-inoculated group were challenged with 10^6^ TCID_50_ OROV wild-type BeAn19991 subcutaneously. The other sham-inoculated group was mock-injected. Mice were followed for weight loss and temperature variation once per day for 7 days following challenge. All injections with virus were performed under anesthesia of 1.5% isoflurane.

### WT OROV serum and organ sampling.

Serum from animals was collected by retrieving blood from the caudal vein. Total blood was centrifuged at 2500 × *g* for 5 min, and clear serum was collected. Seven days after challenge, mice were sacrificed and perfused through the ascending aorta with sterile PBS (pH 7.4). Brain and blood were collected under aseptic conditions.

### RT qPCR.

Tissues were dissected, weighed, and homogenized in sterile PBS using a TissueLyser II instrument (Qiagen). For viral RNA quantification, samples were extracted using a QIAamp viral RNA minikit (Qiagen) according to the manufacturer’s recommendations. Reverse transcription was performed using random primers and the high-capacity reverse transcriptase kit (Thermo Fisher) according to manufacturer’s instructions. Primers were designed to detect a 100-bp region of OROV nucleocapsid from the small segment (S-OROV-reverse: 5′-TTGCGTCACCATCATTCCAA-3′; S-OROV-forward: 5′-TACCCAGATGCGATCACCAA-3′), using SYBR green (Kappa Biosystems). Blood samples were normalized to total volume extracted, and brain samples were normalized to the internal control HRPT1. The reaction was performed in the StepOnePlus PCR system (Applied Biosystems). Standard curves were generated using a plasmid containing the entire OROV small-segment antigenome (pTVT-S described in [3]). Each sample was assayed in duplicate, and the mean value was plotted (3).

### Statistical analysis.

Differences in particle sizes and between VSV and VSV-OROV sera or VSV-OROVΔ4 sera were analyzed using an unpaired *t* test. Body weight and temperature in mouse challenge experiments were compared using a two-way analysis of variance (ANOVA). Differences in brain virus titers were analyzed using the Kruskal-Wallis test. All analysis was performed with GraphPad Prism.

### Animal use and ethical statement.

All VSV recombinant animal experiments and housing were conducted in accordance with protocols approved by the Harvard Medical Area Standing Committee on Animals. All WT OROV studies were approved by the University of São Paulo Committee on Care and Use of Laboratory Animals (protocol no. 194/2019). The 6-week-old C57BL/6 mice were obtained from the Central Animal Facility of the University of São Paulo, School of Medicine, in Ribeirão Preto, SP, Brazil. Infected animals were maintained in the Virology Research Center-FMRP USP animal facility. All animals were kept in accordance with guidelines of the University of São Paulo Committee on Care and Use of Laboratory Animals.
